# The role of physiotherapy interventions in the management of temporomandibular joint ankylosis: a systematic review and meta-analysis: Running title: Physiotherapy in TMJ ankylosis

**DOI:** 10.1186/s13005-024-00416-2

**Published:** 2024-02-29

**Authors:** Ravinder S. Saini, Mohammed Ibrahim, Mohasin Abdul Khader, Masroor Ahmed Kanji, Seyed Ali Mosaddad, Artak Heboyan

**Affiliations:** 1 Department of Dental Technology, COAMS, King Khalid University, Abha, Saudi Arabia; 2 Department of Oral Surgery, College of Dentistry, King Khalid University, Abha, Saudi Arabia; 3 Department of Periodontics and Community Dental Sciences, College of Dentistry, King Khalid University, Abha, Saudi Arabia; 4 Department of Research Analytics, Saveetha Dental College and Hospitals, Saveetha Institute of Medical and Technical Sciences, Saveetha University, Chennai, India; 5 Student Research Committee, School of Dentistry, Shiraz University of Medical Sciences, Qasr-E-Dasht Street, Shiraz, Iran; 6 Department of Prosthodontics, Faculty of Stomatology, Yerevan State Medical University after Mkhitar Heratsi, Str. Koryun 2, 0025 Yerevan, Armenia; 7 Department of Prosthodontics, School of Dentistry, Tehran University of Medical Sciences, Tehran, Iran

**Keywords:** Ankylosis, Physiotherapy, Temporomandibular joint, TMJ Disorders

## Abstract

**Background:**

The main aim of this systematic review and meta-analysis was to identify peer-reviewed scholarly journal articles reporting the significance of physiotherapy interventions in managing TMJ ankylosis. In addition, this study aimed to critically appraise the existing evidence on the prevalence and clinical presentation, physiotherapy intervention approaches, efficacy of physiotherapy interventions, adverse effects, and safety of physiotherapy interventions in TMJ ankylosis management.

**Methods:**

An all-inclusive literature search was conducted using the PubMed, Google Scholar, and Scopus electronic databases. The researchers screened the potential articles and assessed for eligibility based on the reported inclusion and exclusion criteria. The quality evaluation tool for observational cohort and cross-sectional studies developed by the National Institutes of Health (NIH) and the Cochrane Collaboration's Risk of Bias Tool were used to assess the quality of the included studies. Researchers also comprehensively analyzed the data, reported the results, and discussed them according to the predominant themes.

**Results:**

The primary electronic database search yielded 409 articles, of which 25 were included in this review. A secondary search was conducted from citations of the included studies, yielding 74 articles, of which six were included in the study. A significantly higher prevalence of bony ankylosis than fibrous ankylosis, with an overall effect size of *p* < 0.00001. In addition, there were significantly more unilateral than bilateral presentations with an overall effect size of *p* < 0.00001. Moreover, there were 78 reported complications out of 245 subjects according to five included studies demonstrating a significant effect size with *p* = 0.001 following the treatment protocols.

**Conclusion:**

This study highlighted the prevalence of bony ankylosis in temporomandibular joint ankylosis, emphasizing its impact on patients' well-being. On the other hand, the results show that physiotherapy is essential to optimize postoperative outcomes and minimize adverse events such as re-ankylosis. Practitioners and healthcare professionals must monitor postoperative recovery and ensure strict adherence to physiotherapy protocols for optimal outcomes.

**Supplementary Information:**

The online version contains supplementary material available at 10.1186/s13005-024-00416-2.

## Background

Temporomandibular joint (TMJ) ankylosis is a complex and debilitating disorder characterized by the TMJ's abnormal fusion or limited range of motion [1]. This condition significantly affects essential mandibular functions [2]. The predominant etiology of this pathological amalgamation is commonly attributed to the formation of fibrous or osseous adhesions, which hinder the physiological functioning of the joint. TMJ Ankylosis has considerable implications for afflicted individuals, significantly affecting their oral health, dietary intake, and overall quality of life [3, 4].

The etiology of TMJ ankylosis is multifactorial, encompassing various variables such as trauma, infection, inflammation, and congenital anomalies [5]. Injuries, such as fractures affecting the mandible or temporal bone, can disrupt the complex joint structure and trigger a reparative mechanism that results in abnormal adhesions [6]. Infections, regardless of origin from trauma or secondary sources, can trigger inflammatory processes that lead to tissue damage and subsequent ankylosis [7, 8]. Although congenital anomalies are less common, they can heighten an individual's vulnerability to TMJ Ankylosis, presenting challenges in promptly identifying and treating this problem.

TMJ ankylosis clinically manifests as bony or fibrous ankylosis. Bony ankylosis occurs when bones in a joint fuse abnormally, forming osseous tissues and stiff links. Fibrous ankylosis is caused by abundant fibrous connective tissue that restricts joint mobility [6].

Diagnosing ankylosis involves evaluating the extent of adhesions, age at which the problem manifests, etiology, and duration of the ailment. Consequently, effective care of TMJ ankylosis necessitates a comprehensive approach encompassing surgical techniques, orthodontic modalities, and physiotherapeutic procedures [9].

Physiotherapy, a crucial component of this interdisciplinary approach, offers a variety of therapeutic interventions to improve jaw mobility, alleviate pain, and strengthen muscle strength [10, 11]. The interventions involve a variety of therapeutic exercises, manual techniques, and modalities such as heat, cold, or electrical stimulation [12, 13]. The primary goal of personalized physiotherapy interventions is to optimize oral function and alleviate the limitations associated with TMJ ankylosis, ultimately improving overall well-being [10].

There is a notable deficiency in our understanding of the efficacy of physiotherapy in managing TMJ ankylosis. Therefore, this study comprehensively analyzed the existing literature for evidence of the role of physiotherapy in the management of TMJ ankylosis. This research will provide insights into the prevalence and manifestations of TMJ ankylosis. Additionally, it seeks to investigate various physiotherapy intervention approaches, assess their efficacy, and evaluate the associated safety concerns and potential adverse consequences linked to these interventions.

The study's main aim is to emphasize the role of physiotherapy interventions by critically appraising the existing evidence on the prevalence and clinical presentation, physiotherapy intervention approaches, the efficacy of physiotherapy interventions, adverse effects, and safety of physiotherapy interventions in TMJ ankylosis management.

## Materials and methods

The methods and results of this investigation are presented per the Preferred Reporting Items for Systematic Reviews and Meta-Analyses (PRISMA) statement [14]. The protocol for this systematic review was registered at the International Platform of Registered Systematic Review and Meta-Analysis Protocols (INPLASY) (2023100029). The main research question was, "What is the efficacy of physiotherapy interventions in individuals with TMJ Ankylosis?".

### Search strategy

A comprehensive electronic database search up to 8 October 2023 was conducted independently by two reviewers (SAM and AH) to identify peer-reviewed literature published in scholarly journals that reported on the role of physiotherapy interventions in managing TMJ ankylosis. Scopus, Google Scholar, and PubMed were searched using the following search terms: temporomandibular joint ankylosis, TMJ ankylosis, ankylosis of a TMJ, physiotherapy, physical therapy, rehabilitation, exercise therapy, manual therapy, therapeutic exercise, and orofacial exercises.

A secondary search was conducted from the reference lists of the included studies to identify potential articles that reported the significance of physiotherapy interventions in managing TMJ ankylosis.

### Eligibility criteria

This study included current published research on the role of physiotherapy interventions in managing TMJ ankylosis adhering to the PICOS criteria [15]. The included studies reported the prevalence and clinical presentation, physiotherapy intervention approaches, efficacy of physiotherapy interventions, adverse effects, and safety of TMJ ankylosis management. Furthermore, the current investigation encompassed research involving human subjects, specifically those with at least eight participants. Moreover, the present study included reports with access to the full text and available in English. However, Reviews and meta-analyses, reports without methods and results, letters, and editorial notes were excluded from the study.

The PICOS criteria for eligible studies were defined as follows:

- Population (P): Patients diagnosed with TMJ ankylosis.
- Intervention (I): Physiotherapy interventions.
- Comparison (C): No specific comparison is stated in the question.
- Outcome (O): Efficacy in the management of TMJ ankylosis.
- Study Design (S): This study considered Randomized Controlled Trials and other empirical research study designs.

### Data selection and extraction

Article selection was conducted using a procedural screening process. Articles that did not meet the eligibility criteria were omitted from the study based on an assessment of their titles and abstracts. Two reviewers (MI and AK) examined the research titles and abstracts separately, followed by the full text of all papers that fulfilled the eligibility criteria. The reviewers' perspectives were thereafter discussed in order to reach a consensus. All discrepancies or concerns were addressed by engaging a third independent reviewer (MK) and resolved accordingly. Data from the included studies were systematically extracted and double-checked for consistency, as presented in Table 1, including the author, study design, sample size, mean age, study objectives, etiology, clinical presentation, physiotherapy techniques, and study findings.

**Table 1:** Data extraction results

Author	Study Design	Sample Size	Mean age	Study Objectives	Aetiology	Clinical Presentation	Physiotherapy techniques used	Findings
Ahmad et al. (2015)/ [17]	A Prospective Comparative Study	28	Unspecified	To assess modified T-plate interpositional arthroplasty	Trauma, infection, and re-ankylosis	TMJ ankylosis causes facial deformities and reduced mouth opening. Reduced mouth opening causes malnutrition	One day after surgery, active physiotherapy began. Wooden spoons measured mouth openings and physiotherapy efficacy	The modified T-plate interpositional arthroplasty approach is practical in managing TMJ ankylosis
Bayat et al. (2009)/ [18]	Retrospective study	34	21.5	To evaluate gap and interpositional arthroplasty with temporalis muscle flap for TMJ ankylosis	Trauma and osteochondroma	Bony ankylosis	Physiotherapy is an essential part of the treatment At least six months of physiotherapy is recommended. Therabite device	Gap and interpositional arthroplasty using the temporalis muscle flap effectively manage TMJ ankylosis
Braimah et al. (2018)/ [19]	Retrospective study	36	13.8 ± 6.6	To evaluate the TMJ ankylosis management approach	Trauma due to a fall	Bony and fibrous ankylosis	Effective therapy requires intensive postoperative physiotherapy for six months. Preventing re-ankylosis requires jaw physiotherapy	Active physiotherapy is essential for managing TMJ ankylosis
Dowgierd et al. (2022)/ [20]	Single-center prospective cohort study	33	14.24 ± 3.23	To outline TMJ ankylosis treatment	Inflammatory, trauma, and congenital or iatrogenic	Early intervention approach for temporomandibular ankylosis in children and adolescents using 3D virtual surgical planning and customized biomaterials	Before temporomandibular prosthesis insertion, intensive physiotherapy improves mandible function	Gap arthroplasty and thorough therapy before temporomandibular prosthesis outweighed costochondral autografts
Elgazzar et al. (2010)/ [35]	Clinical retrospective study	101	19.43	To explore the experience of managing TMJ ankylosis and compare the outcomes of different protocols	Trauma, previous TMJ surgery, osteoarthritis, hyperplasia, and infection	Bony, fibrous, and ankylosis	- Physiotherapy was a vital part of the treatment - Patients were encouraged to continue mouth-opening exercises at home. massage, and deep heat therapy	Timely TMJ ankylosis release, bone grafting during ramus height reconstruction, and vigorous physiotherapy are efficient management approaches for TMJ ankylosis
Erol et al. (2006)/ [36]	Clinical study	59	18 ± 6.4	To explore the experience of managing TMJ ankylosis	Otitis media, Rheumatoid Arthritis, landslide, traffic accident, birth forceps trauma, and falls	Bony and fibrous ankylosis	Physiotherapy helps avoid postoperative adhesions and re-ankylosis.—Start post-op exercises and physiotherapy immediately	- Falls were the most common cause of ankylosis.—Early postoperative exercises and physiotherapy are essential
Fariña et al. (2018)/ [37]	Clinical study	15	11.4	To establish a treatment approach for TMJ ankylosis emphasizing functional and morphological efficacy	Unspecified	TMJ ankylosis leads to functional and morphological deficits and stunted craniofacial development	- Physiotherapy is fundamental for the stability of treatment results.—It consists of specific exercises performed multiple times a day	The proposed algorithm is functionally and morphologically efficient in managing TMJ ankylosis
Güven O (2000)/ [38]	A clinical and retrospective study	42	Unspecified	To explore the historical background of TMJ ankylosis management	Trauma and Infection	Unilateral ankylosis: mandible hypoplasia, chin deviation on the affected side Bilateral ankylosis: severe retrognathia, mandibular alveolar protrusion, open-bite deformity, bird-face look, hypertrophic and thick coronoid process, night snoring, OSA	- Physiotherapy was used as part of the treatment protocol.—Physiotherapy was reported to be painful Mouth opening and closing exercises using wooden gags and an inter-insical acrylic gag with a jack screw	The spherical acrylic spacer offers a shorter operating time and is economical
Hegab A. F (2015)/ [39]	A Prospective Clinical Study	14	18.5 median age (12—38)	To investigate the efficacy of ankylosis management using pathogenesis	Trauma and falls	- Patients with TMJ ankylosis - Preoperative assessments included patient history, clinical and radiologic examinations	- Wooden tongue blades used - Immediate, continuous aggressive physiotherapy for six months.—Physiotherapy helps prevent adhesions and redevelop muscle function	The treatment protocol is efficient in managing TMJ ankylosis and preventing re-ankylosis
Jain et al. (2008)/ [21]	Retrospective study	44	13.814	To explore TMJ ankylosis management protocols	Falls from heights can cause chin trauma and otitis media	Bony and fibrous ankylosis	Ferguson's mouth gag and wooden Tongue blades Physiotherapy after surgery is essential for long-term maintenance.—Postoperative intense jaw physiotherapy for six months	Timely TMJ ankylosis management is critical. In addition, aggressive physiotherapy is essential for long-term postoperative outcomes
Jakhar et al. (2013)/ [40]	Clinical study	90	14	To investigate the significance of condyle and disc retention in ankylosis management	Trauma	- Severely limited mouth opening with mandibular deviation - No palpable condylar movements or joint pain	- Lack of postoperative physiotherapy led to recurrence in 3 patients.—Intensive physiotherapy program started on the third day postoperatively	The condyle and disc preservation effectively manage TMJ ankylosis with various advantages
Kaban et al. (1990)/ [22]	Retrospective study	14	18.33 ± 12.56	To investigate the efficacy of a TMJ ankylosis management protocol	Trauma, ankylosing spondylitis, and osteochondroma	Fibro-osseous ankylosis, fibrous ankylosis, and bony ankylosis	Aggressive physiotherapy is necessary to eliminate adhesions and avoid soft-tissue constriction. The physiotherapy regimen includes heat, massage, ultrasonography, gum chewing, manual stretching, and the Bell Dynamic Jaw Exerciser	The treatment protocol is effective in managing TMJ ankylosis
Khalifa G. A (2018)/ [41]	Prospective observational clinical study	26	16.27 ± 1.48	To assess mouth-opening changes after gap arthroplasty	Unilateral condylar fracture, Bilateral condylar fracture, and Chin trauma	Type I, II, III, and IV	Mouth gags, mouth prop, and chewing gum	Maximum interincisal opening assessment is critical for the timely detection of re-ankylosis
Kohli et al. (2017)/ [23]	A Prospective Comparative Study	22	24.5	To compare condylar reconstruction approaches regarding function and morphology	Unspecified	- Similar mean mouth opening in both groups	Jaw exercises with mouth gag	Sternoclavicular grafts treat TMJ ankylosis better than transport distraction osteogenesis
Lo et al. (2008)/ [42]	Clinical study	19	29.12	To develop and clinically test a TMJ exerciser	Trauma, mandibular, Orthognathic surgery for cleft deformity, and facial fracture	- TMJ hypomobility and trismus - Patients with various causes of TMJ dysfunction	- The new exerciser is a satisfactory device for physiotherapy of TMJ hypomobility and trismus Power screw technique	The maximal incisor opening increased significantly after using the device
Longobardi et al. (2009)/ [24]	Observational Cohort Study	18	31.3	To assess the efficacy of a three-phase treatment protocol for managing TMJ ankylosis	Previous condylar fractures, Caustic burn, Postsurgical scar, Pyogenic infection, Pseudocamptodactylia, and Trauma with loss of substance	- Limitations in oral opening due to ankylosis	Bite block - Physiotherapy is a phase of the treatment protocol - Physiotherapy is challenging to undertake immediately after surgery	The 3-phase treatment protocol is efficient for managing TMJ ankylosis
Nitzan et al. (2012)/ [25]	Retrospective Case Series	13	20	To explore an alternative treatment approach to TMJ ankylosis using computed tomography	Trauma	Condylar fracture	- Treatment includes intensive supervised physiotherapy	The condyle and disc head displacement are efficiently searched using computed tomography Only ankylotic material is accurately removed, retaining the condyle-disc apparatus
Nouman and Hassan (2017)/ [43]	Experimental study	15	Unspecified	To evaluate the efficacy of physiotherapy following TMJ ankylosis surgery	Unspecified	- TMJ ankylosis interferes with chewing, speech, and oral hygiene - It can cause gross facial deformities if not treated	Facial exercises, electrical stimulation, and using an ice cream stick - Mouth opening exercises and electrical stimulation were used - Facial exercises and home exercise programs implemented	Physiotherapy and mouth-opening exercises are essential in managing TMJ ankylosis
Park et al. (2019)/ [44]	Clinical study	9	35.4	To assess the effectiveness of interocclusal splint for physiotherapy in managing TMJ ankylosis	Trauma and infection	Fibrous, bony ankylosis, Chronic osteomyelitis, pseudo ankylosis, and stylohyoid ligament calcification	Interocclusal splint - Physiotherapy helps prevent adhesion and re-ankylosis	Ankylosed mass resection and physiotherapy are essential in managing TMJ ankylosis
Rahman et al. (2020)/ [45]	Clinical and Radiological Study	15	12.6	To evaluate the suitability of dermal fat for reducing pain during active physiotherapy	Fall from height and infection of the ear	Osseous or fibro-osseous ankylosis	Early, intensive postoperative physiotherapy is crucial	The dermis fat graft could be a superior choice in managing TMJ ankylosis
Sahoo et al. (2012)/ [46]	Clinical study	64	14.3	To compare the outcomes of alternative approaches for managing TMJ ankylosis	Trauma, infection, and systemic illness	Limitations in mouth opening, dentofacial deformities, malocclusion, poor oral hygiene, dental caries, aesthetic impairment, malnutrition, and OSA	Ice cream blades - Non-compliance to postsurgical physiotherapy led to re-ankylosis - Active physiotherapy was carried out postoperatively for six months	Interpositional arthroplasty with temporalis myofascial flap is effective for mild mandibular deformities
Sami et al. (2023)/ [26]	Prospective study	12	11.2	To compare the outcomes of using temporalis fascia as an interpositional graft	Fall from height and ear infections	Unspecified	Early, intensive postoperative physiotherapy is crucial Physiotherapy prevents and treats TMJ hypomobility and ankyloses	Cutaneous fat grafts and temporal fascia are effective when treating TMJ ankylosis
Shetty et al. (2019)/ [27]	Retrospective study	98	20	To evaluate the outcomes of a two-phase physiotherapy approach after consecutive ankylotic mass resection	Unspecified	Problems with mastication, talking, and mouth opening (re-ankylosis) are common	- A novel physiotherapy procedure involving two stages was demonstrated - The success of the physiotherapy treatment relies heavily on patient acceptance	The longevity and rigidity of interpositional graft insignificantly influence the outcomes of TMJ ankylosis management
Shivakotee et al. (2020)/ [28]	Case series	18	17.66	To measure the effectiveness of treatments for TMJ ankylosis	Trauma and Congenital	- Mastication, digestion, speech, and hygiene can all be affected by TMJ ankylosis - Common among young children	- Physiotherapy is emphasized for all patients	Interpositional arthroplasty with vascularized temporalis fascia flap can avoid re-ankylosis
Singh et al. (2014)/ [29]	Retrospective study	15	12.2	To assess lateral arthroplasty for TMJ ankylosis	Trauma	- Trauma was the etiological factor in all cases	Mouth prop, mouth gag, and spoon spatulas - Postoperative physiotherapy began on day one The therapy comprised active and passive exercises	Type III ankylosis patients benefit from the medially displaced condyle and disc
Singh et al. (2012)/ [30]	Prospective study	10	17.7	To assess the suitability of sternoclavicular graft as an interposition graft in managing TMJ ankylosis	Trauma and infection	- 10 patients with TMJ ankylosis, aged 12–35 years - Complete osseous ankylosis, mean duration 6.4 years	Mouth prop, mouth gag, and spoon spatulas - The physiotherapy treatment comprised active hinge-opening and excursive movements	Sternoclavicular Graft, Buccal Fat Pad Lining interposition, and active physiotherapy are essential for managing TMJ ankylosis
Tauro and Manay (2020)/ [31]	Observational cohort study	21	19	To propose modifications to the surgical approaches in managing and minimizing re-ankylosis	Unspecified	21 patients with TMJ ankylosis	- Aggressive intermittent intraoperative jaw physiotherapy - Rigorous postoperative jaw physiotherapy	The proposed approach effectively minimizes re-ankylosis
Lin et al. (2019)/ [32]	Retrospective study	32	Unspecified	To explore the outcomes of retaining the medially displaced residual condyle in managing TMJ ankylosis	Accidental impact, violence, and accidental fall	- Limited ability to open mouth, difficulties with eating and speech	- Physiotherapy involves active and passive mandibular movement and maximal mouth-opening exercises	The displaced condyle should be preserved in managing TMJ ankylosis
Yadav et al. (2021)/ [33]	Retrospective study	114	15.75 ± 9.76	To investigate a method for reducing re-ankylosis after TMJ ankylosis surgery	Trauma and infection	- 114 patients (n = 152 joints) evaluated retrospectively - Interpositional arthroplasty, costochondral graft, and complete joint replacement were used	Tapered acrylic trismus screw - Aggressive physiotherapy is vital to prevent re-ankylosis	The risk of re-ankylosis can be minimized by following the proposed treatment protocol
Younis et al. (2020)/ [47]	Prospective Clinical Comparative Study	30	6.5	To compare cutaneous fat graft and temporalis myofascial flap as interposition grafts for TMJ ankylosis	Trauma and otitis media	- TMJ ankylosis causes difficulty in chewing, speech, and oral hygiene	Wooden spatulas - Physiotherapy techniques were used in the study	Dermis fat grafts may be better than temporalis myofascial flaps for treating TMJ ankylosis
Zhang & He (2006)/ [48]	Retrospective study	18	28	To assess condylar fracture-related TMJ ankylosis and postoperative outcomes	Trauma	- Type I ankylosis develops in the 4th to 5th-month post-trauma with 183 ± 55 mm mean interincisal opening	Physiotherapy	Disc repositioning is effective for TMJ ankylosis management - Close follow-up for 18 months after condylar fractures - Surgical intervention for fibrous ankylosis after two months

*TMJ* Temporomandibular joint

### Methodological quality assessment

The 17 prospective, retrospective, and observational studies included in this analysis were assessed using the quality evaluation tool for observational cohort and cross-sectional studies developed by the National Institutes of Health (NIH) [16]. On the other hand, 14 clinical and experimental studies were methodically evaluated using the Cochrane Collaboration's Risk of Bias Tool [34]. The quality assessment process for all selected articles was performed independently by two scorers (RS and AH).

### Data analysis

Data from the included studies were systematically extracted and are presented in Table 1. The results were reported according to the prevalence, clinical presentation, physiotherapy intervention approaches, efficacy of physiotherapy interventions, adverse effects, and safety in TMJ ankylosis management. In addition, an intervention review approach was applied in analyzing quantitative data using Review Manager version 5.4.1. Moreover, proportion meta-analyses were conducted using the random effects analysis approach, the Mantel–Haenszel statistical method, and the odds ratio as the effect measure. A 95% confidence interval was applied in the analyses. The assessment of publication bias was conducted using Egger's test within the Review Manager 5 (RevMan 5) software (Version 5.4. Copenhagen: The Cochrane Collaboration, 2020).

## Results

### Study selection

**Fig. 1:**
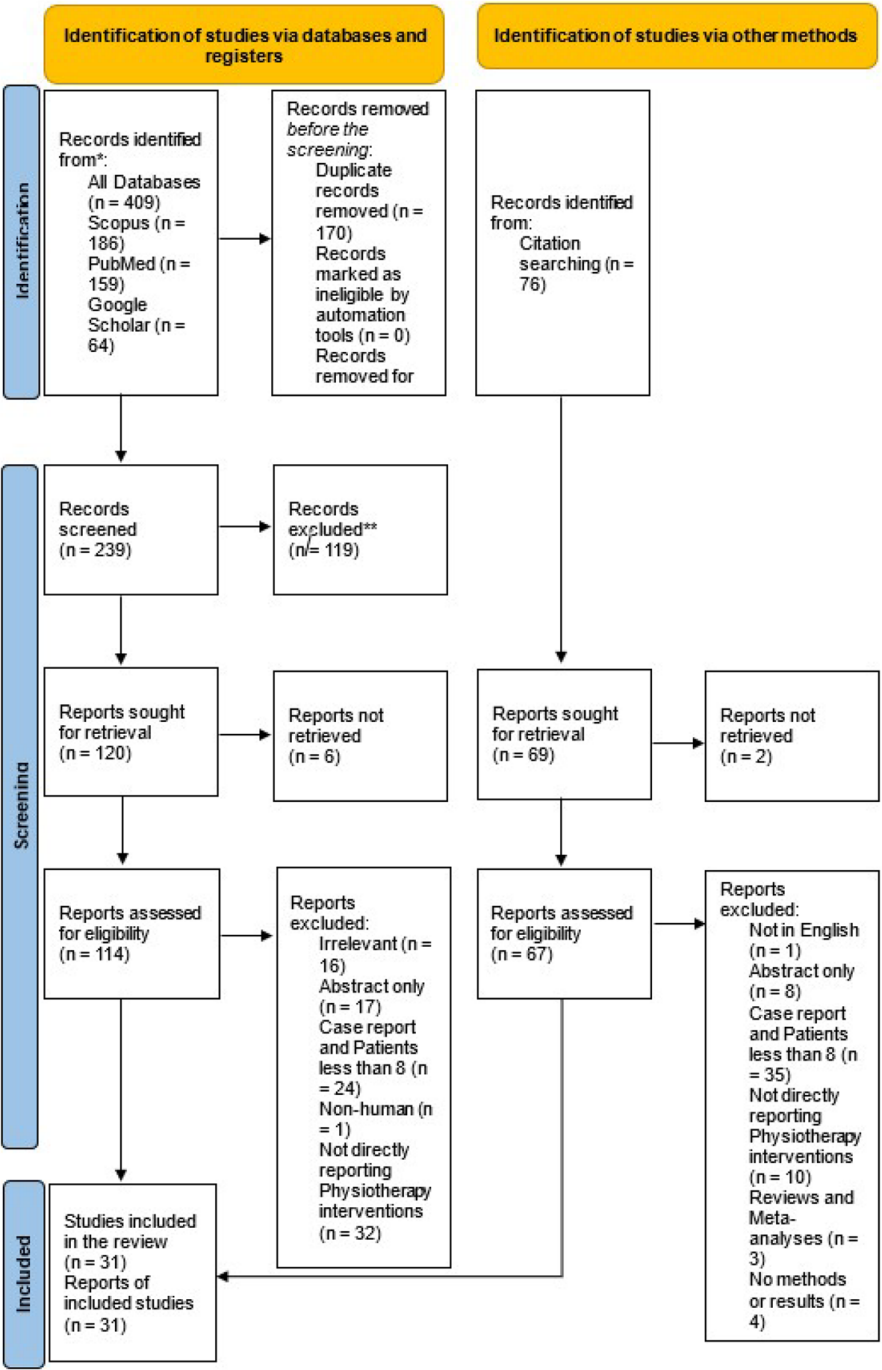
PRISMA flow diagram showing the results of the study selection process

The literature search yielded 409 articles, of which 170 duplicates were removed. After title and abstract screening, 119 articles were excluded. The remaining 120 articles were retrieved, after which 25 studies that met the eligibility criteria were included. In addition, a secondary search of the reference lists yielded 74 articles, of which seven duplicate records were eliminated. Two articles could not be retrieved, and 67 were assessed for eligibility, after which six met the eligibility criteria and were included in the study. Figure 1 displays the obtained data.

### Methodological quality assessment

**Fig. 2:**
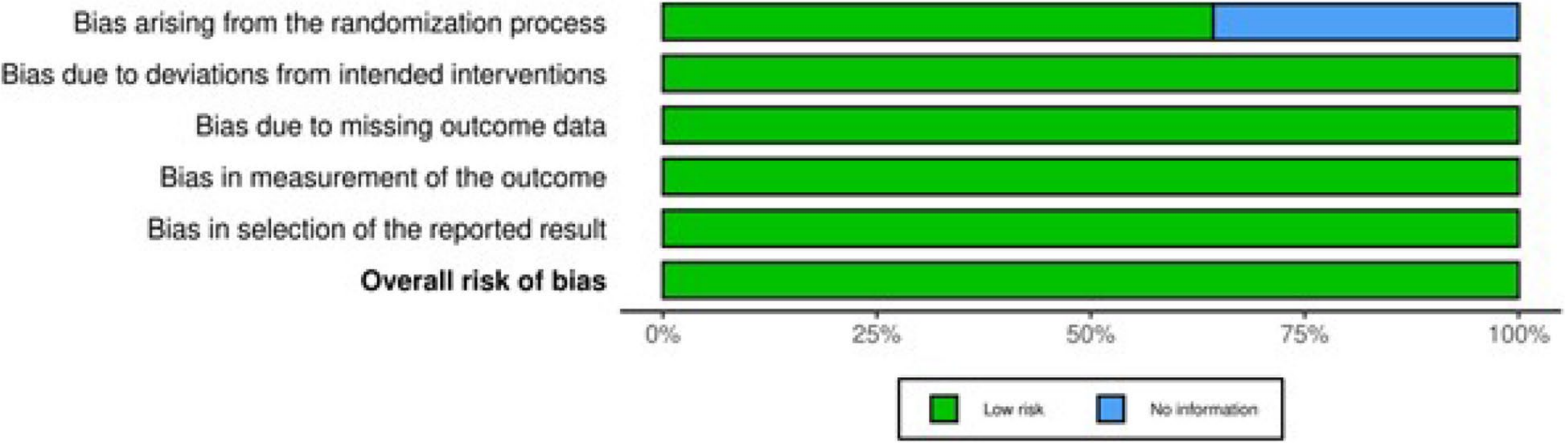
Summary plot showing the results of Cochrane Collaboration's Risk of Bias [34]

**Fig. 3:**
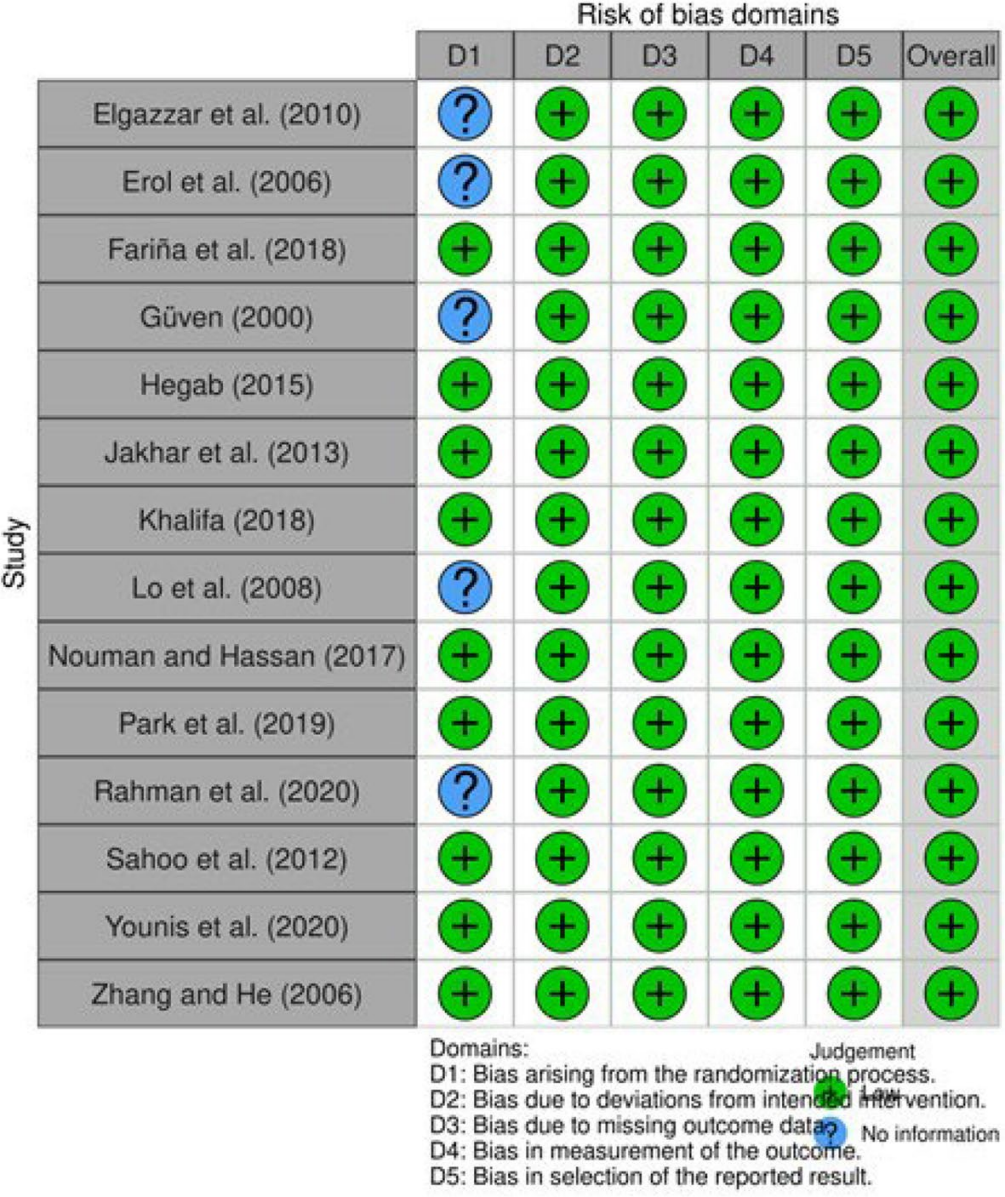
Traffic light plots showing the Cochrane Collaboration's Risk of Bias results [34]

The results of the National Institutes of Health (NIH) quality assessment are presented in Table 2. In addition, Figs. 2 and 3 show the Cochrane Collaboration's Risk of Bias assessment results.

**Table 2:** National Institutes of Health (NIH) quality assessment results [16]

Author	Objectives clearly stated	Well-defined research population	At least 50% of those eligible participate	Participants from similar groups	Justification of sample size, power, variance, and effect estimates	Interest exposures before outcomes	A realistic timeframe to correlate exposure and consequence	Overall rating
Ahmad et al. (2015) [17]	Y	Y	Y	Y	N	Y	Y	G
Bayat et al. (2009) [18]	Y	Y	Y	Y	N	Y	Y	G
Braimah et al. (2018) [19]	Y	Y	Y	Y	N	Y	Y	G
Dowgierd et al. (2022) [20]	Y	Y	Y	Y	N	Y	Y	G
Jain et al. (2008) [21]	Y	Y	Y	Y	N	Y	Y	G
Kaban et al. (1990) [22]	Y	Y	Y	Y	N	Y	Y	G
Kohli et al. (2017) [23]	Y	Y	Y	Y	N	Y	Y	G
Longobardi et al. (2009) [24]	Y	Y	Y	Y	N	Y	Y	G
Nitzan et al. (2012) [25]	Y	Y	Y	Y	N	Y	Y	G
Sami et al. (2023) [26]	Y	Y	Y	Y	N	Y	Y	G
Shetty et al. (2019) [27]	Y	Y	Y	Y	N	Y	Y	G
Shivakotee et al. (2019) [28]	Y	Y	Y	Y	N	Y	Y	G
Singh et al. (2014) [29]	Y	Y	Y	Y	N	Y	Y	G
Singh et al. (2012) [30]	Y	Y	Y	Y	N	Y	Y	G
Tauro and Manay (2020) [31]	Y	Y	Y	Y	N	Y	Y	G
Lin et al. (2019) [32]	Y	Y	Y	Y	N	Y	Y	G
Yadav et al. (2021) [33]	Y	Y	Y	Y	N	Y	Y	G

Y = Yes

N = No

G = Good

### Study characteristics

The results of the data analysis are thematically reported according to the predominant themes: prevalence and clinical presentation of TMJ ankylosis; physiotherapy interventions approach for TMJ ankylosis, the efficacy of physiotherapy interventions in TMJ ankylosis management, adverse effects and safety of physiotherapy interventions [49].

### Prevalence and clinical presentation of TMJ ankylosis

**Fig. 4:**
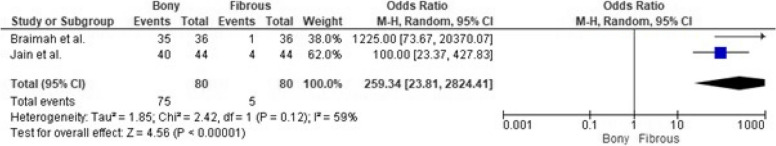
Forest plot comparing the prevalence of bony and fibrous ankyloses [19, 21]

These studies reported quantitative data comparing the clinical manifestations of TMJ ankylosis. Nevertheless, the findings of these two investigations were incorporated into the meta-analysis. A proportion meta-analysis showed a significantly higher prevalence of bony ankylosis than fibrous ankylosis with an overall effect size of *p* < 0.00001 (Fig. 4). The total events were 75 out of 80 subjects, as reported by two of the included studies.

In contrast, Bayat et al. [18] reported that all participants had bony ankylosis. Bony ankylosis was found in 77 (71%), fibrous ankylosis in 6 (6%), and fibro-osseous ankylosis in 26 (24%) patients by Elgazzar et al. [35]. On the other hand, a study by Dowgierd et al. [20] reported that 82% of the joints under study had bony-type ankylosis. The study by Jain et al. [21] had nine out of 18 joints manifesting fibro-osseous ankylosis. Computed tomography scans revealed fibro-osseous in 9 joints, fibrous in 5, and bony ankylosis in 4 [22]. Khalifa [41] investigated bony ankylosis and reported type II manifestation in three patients (11.54), type III in 22 patients (84.61), and type IV in one patient (3.85).

**Fig. 5:**
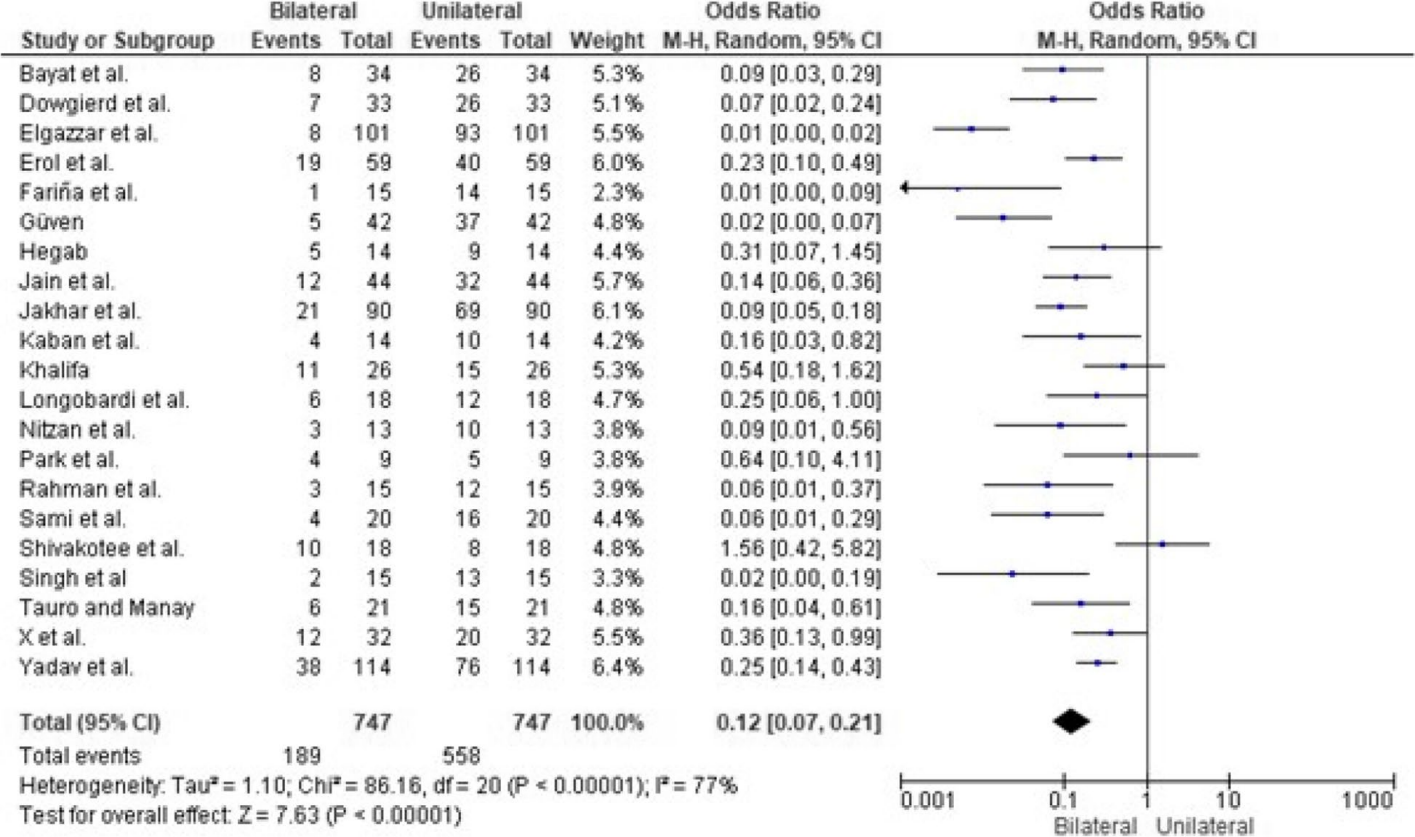
Forest plot comparing the prevalence of bilateral and unilateral presentation of TMJ ankylosis [18, 20–22, 24–26, 28, 29, 31–33, 35, 37–41]

**Fig. 6:**
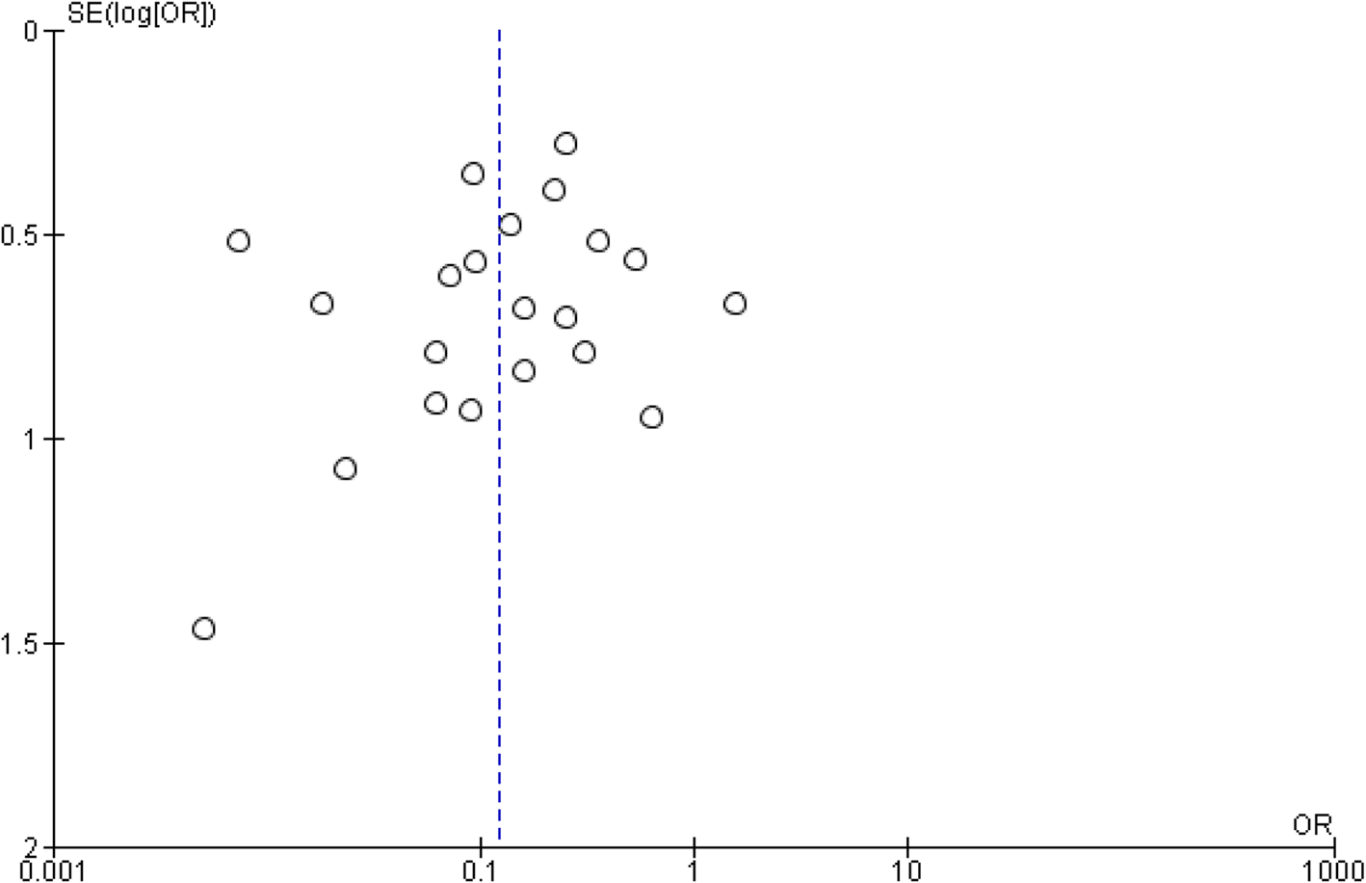
The funnel plot illustrates the publication bias, comparing the prevalence of bilateral and unilateral presentation of TMJ ankylosis [18, 20–22, 24–26, 28, 29, 31–33, 35, 37–41]

There were 189 bilateral events and 558 events out of 747 subjects, as reported by 21 of the included studies, demonstrating significantly more unilateral than bilateral presentations with an overall effect size of *p* < 0.00001, as shown in Fig. 5. Figure 6 shows the publication bias of the clinical manifestation of the temporomandibular joint ankylosis analysis.

### Physiotherapy interventions approaches for TMJ ankylosis

The studies that were included reported active physiotherapy as part of the treatment protocol. Various devices have been used, including wooden spoons [17]. The participants were instructed to utilize wooden spoons positioned between their incisors, gradually increasing the quantity of spoons at predefined intervals. According to Bayat et al. [18], patients used Therabite devices for continuous passive exercise. Elgazzar et al. [35] reported mouth opening and jaw exercises, massage, and deep heat therapy.

Customized mouth gags were used by patients, including inter-incisal acrylic gags with a jack screw, according to Güven et al. [38]. The study participants used wooden tongue blades, increasing the numbers gradually depending on the subjects' tolerance in the study by Hegab [39]. Similarly, a study [21] reported patients using Ferguson's mouth gag and wooden tongue blades for active jaw physiotherapy. According to Kaban et al. [22], physiotherapy was performed using heat, massage, ultrasonography, gum chewing, manual stretching exercises, and a bell dynamic jaw exerciser.

In addition, mouth gags, props, and chewing gum have been used for aggressive physiotherapy [41].

### Efficacy of physiotherapy interventions in TMJ ankylosis management

Ahmad et al. [17] reported the simplicity and ease of measuring mouth opening using wooden spoons, demonstrating physiotherapy's effectiveness in managing TMJ ankylosis. The number of spoons accommodated was used to measure the extent of the mouth opening.

On the other hand, physiotherapy is essential in preventing re-ankylosis [18–21]. Studies by Elgazzar et al. [35] and Lin et al. [32] have reported non-compliance with physiotherapy protocols as a significant cause of postoperative complications. According to Singh et al. [30], physiotherapy is essential for preserving the postoperative outcomes of surgical intervention.

### Adverse effects and safety of physiotherapy interventions

**Fig. 7:**
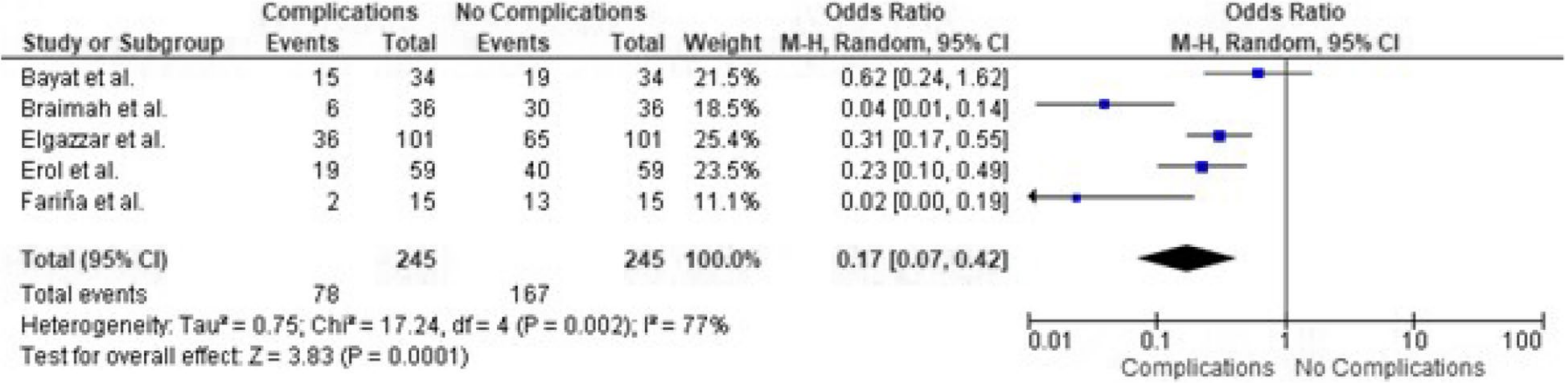
Forest plot showing the complication rates following TMJ ankylosis treatment [18, 19, 35–37]

**Fig. 8:**
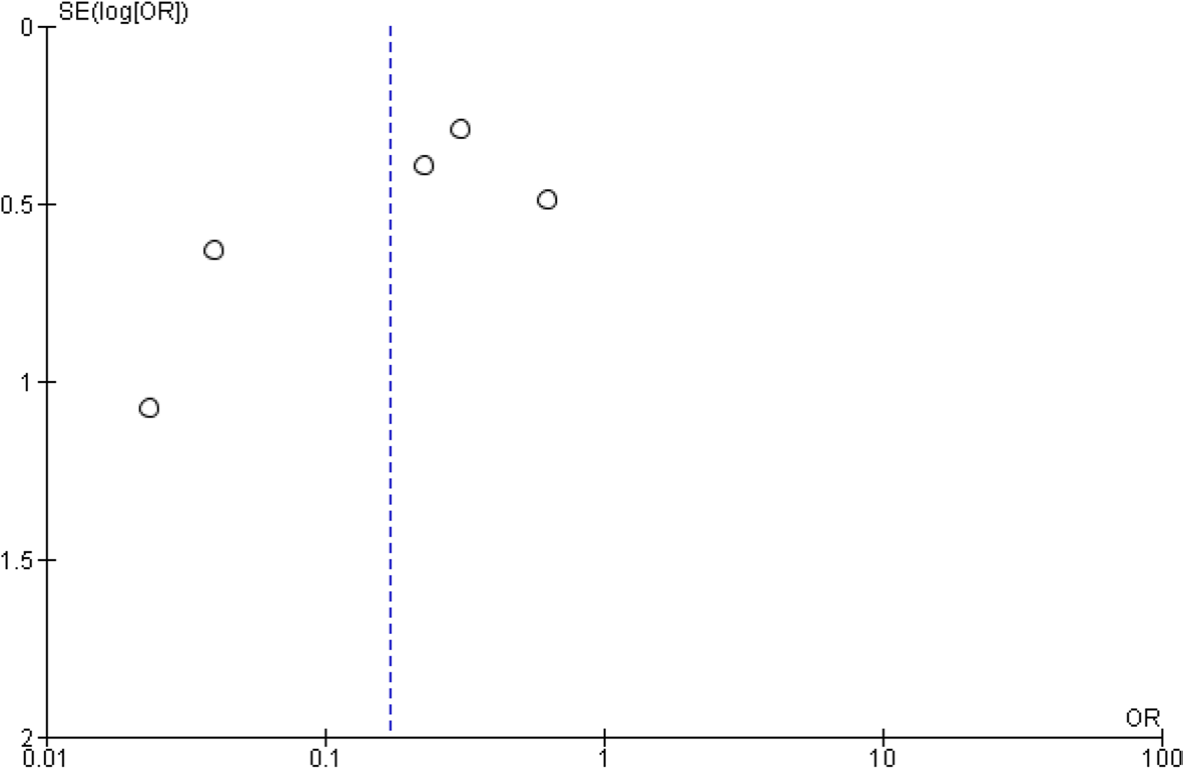
The funnel plot illustrates the publication bias regarding the complication rates following TMJ ankylosis treatment [18, 19, 35–37]

There were 78 reported complications out of 245 subjects according to five included studies demonstrating a significant effect size with *p* = 0.001 following the treatment protocols, as shown in Fig. 7. Figure 8 shows the risk of bias assessment of the safety profile analysis.

Ahmad et al. [17] reported that complication rates were significantly low in modified T-plate interposition arthroplasty, reporting three patients with complications, including hematoma, infection, and re-ankylosis. In contrast, Bayat et al. [18] reported complications, including re-ankylosis, postoperative infection, and temporary facial palsy. Some patients had a severe hemorrhage, facial nerve palsy, and re-ankylosis [19].

According to Dowgierd et al. [20], six patients reported complications, including ectopic bone formation on the TMJ in one patient, relapse in three patients, and infection in two patients. In addition, Elgazzar et al. [35] reported persistent pain, limited mouth opening, facial weakness, partial graft resorption, intraoperative bleeding, slight condylar overgrowth, transient facial nerve weakness, loose hardware, infection, and re-ankylosis. Patients reported temporary paresis of the facial nerve, open bite, re-ankylosis, and Frey's syndrome [36].

Patients reported pain during physiotherapy in the early postoperative days [26, 39, 45, 47]. Excessive bleeding, wound hematoma, re-ankylosis, facial nerve injury, and wound infections have been reported by patients [21]. Conversely, no complications have been observed [26, 28, 39, 42]. According to Park et al. [44], one patient reported an anterior open bite.

## Discussion

TMJ ankylosis manifests clinically in various ways. The study results showed that bony ankylosis is more common than fibrous ankylosis. Bony ankylosis is characterized by stiffness, limiting joint movements, and significantly affecting the patient's general health [50]. In addition, most patients reported unilateral manifestations rather than bilateral cases. Understanding the clinical manifestations is critical for developing effective treatment protocols for managing TMJ ankylosis while minimizing recurrence and adverse effects.

Higher bony ankylosis prevalence compared to fibrous may be associated with various factors. Bony-type ankylosis may result from more severe inflammatory processes or trauma, leading to increased clinical manifestations and patient reporting. In addition, bony ankylosis is more visible diagnostically, manifesting in the form of joint stiffness, thus easily identifiable, contributing to its higher reported prevalence in the included studies. Moreover, the adverse effect of bony ankylosis on the patient's quality of life prompts diagnosis and intervention, which are more prevalent in clinical studies.

In addition, the results show various treatment protocols incorporating different physiotherapy techniques to optimize the postoperative results of TMJ ankylosis. Physiotherapy techniques use multiple tools, including wooden spoons, Therabite devices, personalized mouth gags, and various exercises [17, 18]. Physiotherapy techniques optimize mouth opening and accelerate healing after surgery. The included studies emphasized the significance of physiotherapy in minimizing re-ankylosis and other postoperative complications.

On the other hand, most physiotherapy techniques were considered safe and effective in minimizing adverse events [18–21, 27]. There were considerably fewer postoperative adverse events, as shown in Fig. 6. However, pain during physiotherapy has been reported. Nevertheless, the pain reduced over time, gradually making it easy to continue physiotherapy. In addition, evidence shows that physiotherapy therapies are essential for optimizing postoperative outcomes and preventing re-ankylosis. The devices employed, such as wooden spoons in the treatment protocols, are simple to use and can be used to measure mouth opening and keep track of progress. These studies emphasize compliance with physiotherapy protocols for optimal results.

The incidence of postoperative complications was significantly lower. Nevertheless, understanding the possible adverse events of physiotherapy treatments is vital for providing complete patient care. Therefore, it is essential to carefully monitor patients after surgery and emphasize compliance with predefined protocols to minimize hematomas, infections, facial nerve palsy, and re-ankylosis.

The physiotherapy protocols have a relatively good reported safety and compliance due to the customized and gradual nature of specific individual needs. Appropriate protocols emphasize patient comfort, enhancing compliance while minimizing adverse effects and postoperative complications. In addition, simple tools used and regular monitoring strengthen the safety of the treatment protocols.

This comprehensive analysis of the clinical manifestations and treatment protocols for TMJ ankylosis highlights the importance of understanding the various aspects of this condition. Bony ankylosis, more prevalent than fibrous ankylosis, can significantly impact the patient's quality of life. Incorporating physiotherapy techniques into the treatment plans is crucial for optimizing postoperative outcomes, minimizing re-ankylosis, and reducing complications.

While physiotherapy techniques have proven safe and effective, healthcare professionals should remain vigilant in monitoring patients after surgery and ensuring compliance with predefined protocols. This proactive approach can help minimize adverse events and provide complete patient care. By continuing to advance our knowledge of the clinical manifestations and treatment options for TMJ ankylosis, we can improve patient outcomes and enhance the overall management of this condition.

### Limitations of the evidence included in the review

While the evidence presented in the previous discussion provides valuable insights into the clinical manifestations and treatment protocols for TMJ (TMJ) ankylosis, it is essential to acknowledge the limitations of the reviewed studies. These limitations may affect the generalizability and reliability of the findings.

1. Small Sample Size: Some of the included studies in the analysis had a small sample size, which could impact the statistical power and generalizability of the results.
2. Heterogeneity of Study Designs: The studies encompassed a range of designs, leading to heterogeneity in methodology and outcome measurements. This variance in study designs may make it challenging to draw definitive conclusions or establish standardized treatment protocols for TMJ ankylosis.
3. Lack of Randomization and Blinding: Several studies did not employ randomization or blinding techniques, which raises the potential for bias in the results. Without randomization, there is an increased risk of selection bias, as participants may not be representative of the overall population or the specific subgroups being studied.
4. Lack of Long-term Follow-up: Many of the included studies had a relatively short follow-up period, which limits the ability to assess the long-term effectiveness and potential complications associated with the treatment protocols.

It is crucial to consider these limitations when interpreting the evidence presented. Further research with larger sample sizes, standardized study designs, randomized controlled trials, and long-term follow-up periods is needed to address these limitations and provide more robust evidence on the clinical manifestations and treatment protocols for TMJ ankylosis.

### Implications for clinical practice

The findings presented in the above article have several implications for clinical practice in the management of TMJ (TMJ) ankylosis:

- 1. Treatment Protocol Optimization: Clinicians should consider incorporating physiotherapy techniques, such as wooden spoons, Therabite devices, personalized mouth gags, and specific exercises, to improve postoperative outcomes and minimize the chances of re-ankylosis.
- 2. Patient Compliance and Education: Healthcare professionals should educate patients about the benefits of physiotherapy and the potential consequences of non-compliance, including re-ankylosis and other postoperative complications.
- 3. Risk and Complication Management: Healthcare practitioners should be vigilant in monitoring patients after surgery, mainly to prevent complications such as hematomas, infections, facial nerve palsy, and re-ankylosis. Adherence to predefined protocols for postoperative care is crucial in minimizing the incidence of adverse events. Regular follow-up appointments are essential to evaluate the long-term outcomes and address emerging complications.

### Implications for policy

The research findings have several implications for healthcare policies related to TMJ ankylosis:

- 1. Standardized Treatment Guidelines: Policymakers should consider developing standardized treatment guidelines for TMJ ankylosis. These guidelines should include recommendations for incorporating physiotherapy techniques as an integral part of the treatment process.
- 2. Access to Physiotherapy Services: Policymakers should evaluate the availability and accessibility of physiotherapy services for patients with TMJ ankylosis. Ensuring that these services are readily available to patients, regardless of geographical location or financial barriers, would support optimal postoperative outcomes and reduce the likelihood of complications or re-ankylosis.

### Implications for future research

While the discussed study contributes to the understanding of TMJ ankylosis, there are opportunities for further research:

- 1. Randomized Controlled Trials: Future research should focus on conducting well-designed randomized controlled trials with larger sample sizes. These trials would provide a higher level of evidence on the effectiveness and safety of physiotherapy techniques for TMJ ankylosis.
- 2. Long-term Follow-up Studies: Longitudinal studies with extended follow-up periods are needed to evaluate the sustained outcomes of various treatment protocols for TMJ ankylosis. These studies would provide insights into the long-term functional improvements, rates of recurrence, and potential complications associated with different treatment approaches.
- 3. Comparative Effectiveness Research: Comparative effectiveness research comparing various physiotherapy techniques and treatment approaches would help determine the most effective interventions for TMJ ankylosis. This research would assist clinicians in making informed decisions regarding selecting treatment protocols for individual patients.

## Conclusion

The present study evaluated the prevalence and clinical presentation of physiotherapy intervention approaches, efficacy of physiotherapy interventions, adverse effects, and safety of physiotherapy interventions in TMJ ankylosis management. This study highlighted the prevalence of bony ankylosis in temporomandibular joint ankylosis, emphasizing its impact on patients' well-being. On the other hand, the results show that physiotherapy is essential to optimize postoperative outcomes and minimize adverse events such as re-ankylosis. Practitioners and healthcare professionals need to monitor postoperative recovery and ensure strict adherence to physiotherapy protocols for optimal outcomes. However, there is limited empirical research directly investigating the role of physiotherapy interventions in managing TMJ ankylosis. Therefore, further studies should be carried out to verify the results of this study.

### Supplementary Information

**Supplementary Material 1.**

## Data Availability

No datasets were generated or analysed during the current study.
